# Poly[bis­[μ_2_-8-ethyl-5-oxo-2-(piperazin-1-yl)-5,8-dihydro­pyrido[2,3-*d*]pyrimidine-6-carboxyl­ato]nickel(II)]

**DOI:** 10.1107/S1600536809055408

**Published:** 2010-01-09

**Authors:** Zhe An, Ling Zhu

**Affiliations:** a School of Chemistry and Life Science, Maoming University, Maoming 525000, People’s Republic of China

## Abstract

The title compound, [Ni(C_14_H_16_N_5_O_3_)_2_]_*n*_ or [Ni(ppa)_2_]_*n*_, where ppa is 8-ethyl-5-oxo-2-(piperazin-1-yl)-5,8-dihydro­pyrido[2,3-*d*]pyrimidine-6-carboxyl­ate, was synthesized under hydro­thermal conditions. The Ni^II^ atom (site symmetry 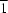) exhibits a distorted *trans*-NiN_2_O_4_ octa­hedral geometry defined by two monodentate *N*-bonded and two bidentate *O*,*O*′-bonded ppa monoanions. The extended two-dimensional structure is a square grid. An inter­molecular N—H⋯O hydrogen bond occurs.

## Related literature

For manganese, cobalt and zinc complexes of the ppa anion, see: Huang *et al.* (2008 ▶); Xu *et al.* (2009 ▶); Qi *et al.* (2009 ▶), respectively. For background to the medicinal uses of pipemidic acid, see: Mizuki *et al.* (1996 ▶).
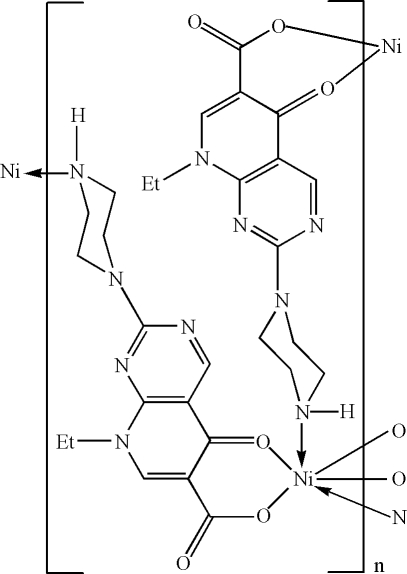


## Experimental

### 

#### Crystal data

[Ni(C_14_H_16_N_5_O_3_)_2_]
*M* *_r_* = 663.35
Monoclinic, 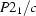

*a* = 6.1249 (6) Å
*b* = 21.250 (2) Å
*c* = 12.5511 (12) Å
β = 101.846 (2)°
*V* = 1598.8 (3) Å^3^
*Z* = 2
Mo *K*α radiation
μ = 0.66 mm^−1^
*T* = 296 K
0.43 × 0.28 × 0.22 mm

#### Data collection

Bruker APEXII CCD diffractometer
Absorption correction: multi-scan (*SADABS*; Bruker, 2004 ▶) *T*_min_ = 0.764, *T*_max_ = 0.868
7593 measured reflections
2762 independent reflections
2389 reflections with *I* > 2σ(*I*)
*R*_int_ = 0.034

#### Refinement

*R*[*F* ^2^ > 2σ(*F* ^2^)] = 0.066
*wR*(*F* ^2^) = 0.199
*S* = 1.00
2762 reflections
209 parameters
1 restraint
H-atom parameters not refined
Δρ_max_ = 0.89 e Å^−3^
Δρ_min_ = −0.39 e Å^−3^

### 

Data collection: *APEX2* (Bruker, 2004 ▶); cell refinement: *SAINT-Plus* (Bruker, 2004 ▶); data reduction: *SAINT-Plus*; program(s) used to solve structure: *SHELXS97* (Sheldrick, 2008 ▶); program(s) used to refine structure: *SHELXL97* (Sheldrick, 2008 ▶); molecular graphics: *SHELXTL* (Sheldrick, 2008 ▶); software used to prepare material for publication: *SHELXTL*.

## Supplementary Material

Crystal structure: contains datablocks I, global. DOI: 10.1107/S1600536809055408/hb5292sup1.cif

Structure factors: contains datablocks I. DOI: 10.1107/S1600536809055408/hb5292Isup2.hkl

Additional supplementary materials:  crystallographic information; 3D view; checkCIF report

## Figures and Tables

**Table 1:** Selected bond lengths (Å)

Ni1—O2	2.013 (3)
Ni1—O1	2.051 (3)
Ni1—N5^i^	2.207 (3)

Symmetry code: (i) 
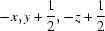
.

**Table 2:** Hydrogen-bond geometry (Å, °)

*D*—H⋯*A*	*D*—H	H⋯*A*	*D*⋯*A*	*D*—H⋯*A*
N5—H5*N*⋯O3^ii^	0.90 (4)	2.29 (4)	3.161 (5)	163 (5)

Symmetry code: (ii) 
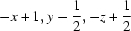
.

**Fig. 1.**
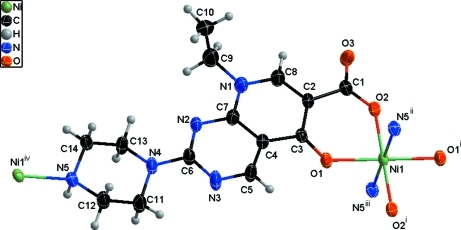
The asymmetric unit of (I), expanded to show the metal atom coordination showing 50% displacement ellipsoids.

**Fig. 2.**
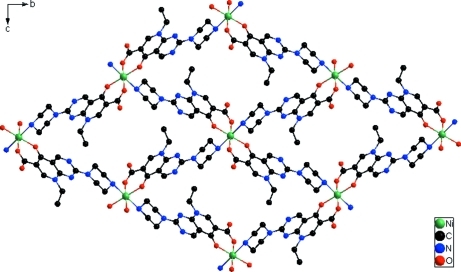
A view of part of a two-dimensional polymeric sheet in (I) showing the square-grid connectivity.

## supplementary crystallographic information

### Comment

Pipemidic acid (Hppa, C_14_H_16_N_5_O_3_, 8-Ethyl-5,8-dihydro-5-oxo-2- (1-piperazinyl)-pyrido(2,3-d)-pyrimidine-6-carboxylic acid) is member of a class of quinolones used to treat infections (Mizuki *et al.*, 1996). The manganese, cobalt and zinc complexes of the ppa anion have been reported (Huang *et al.*, 2008; Xu *et al.* 2009; Qi Xu *et al.*2009). The title nickel(II) complex, (I), is reported here (Fig. 1).

The nickel(II) atom is coordinated by four oxygen atoms and two N atoms from four ppa ligands (two monodentate-N and two O,*O*-bidentate) to form a square grid propagating in (Fig. 2).

### Experimental

A mixture of Ni(CH_3_COO)_2_.4H_2_O (0.063 g, 0.25 mmol), Hppa (0.15 g, 0.5 mmol), sodium hydroxide (0.04 g, 1 mmol) and water (15 ml) was stirred for 30 min in air. The mixture was then transferred to a 25 ml Teflon-lined hydrothermal bomb. The bomb was kept at 443 K for 72 h under autogenous pressure. Upon cooling, green prisms of (I) were obtained from the reaction mixture.

### Refinement

The carbon-bound H atoms were positioned geometrically (C—H = 0.93–0.97 Å) and were included in the refinement in the riding model approximation, with U(H) = 1.2Ueq(C). The H on Nitrogen atoms were located in a difference Fourier map, and were refined with a distance restraint of N—H = 0.86 (1) /%A and with *U*_iso_(H) = 1.2Ueq(N).

### Crystal data

**Table:**

[Ni(C_14_H_16_N_5_O_3_)_2_]	*F*(000) = 692
*M**_r_* = 663.35	*D*_x_ = 1.378 Mg m^−^^3^
Monoclinic, *P*2_1_/*c*	Mo *K*α radiation, λ = 0.71073 Å
Hall symbol: -P 2ybc	Cell parameters from 3258 reflections
*a* = 6.1249 (6) Å	θ = 2.5–28.3°
*b* = 21.250 (2) Å	µ = 0.66 mm^−^^1^
*c* = 12.5511 (12) Å	*T* = 296 K
β = 101.846 (2)°	Prism, green
*V* = 1598.8 (3) Å^3^	0.43 × 0.28 × 0.22 mm
*Z* = 2	

### Data collection

**Table:**

Bruker APEXII CCD diffractometer	2762 independent reflections
Radiation source: fine-focus sealed tube	2389 reflections with *I* > 2σ(*I*)
graphite	*R*_int_ = 0.034
φ and ω scans	θ_max_ = 25.1°, θ_min_ = 2.5°
Absorption correction: multi-scan (*SADABS*; Bruker, 2004)	*h* = −7→7
*T*_min_ = 0.764, *T*_max_ = 0.868	*k* = −25→23
7593 measured reflections	*l* = −14→9

### Refinement

**Table:**

Refinement on *F*^2^	Primary atom site location: structure-invariant direct methods
Least-squares matrix: full	Secondary atom site location: difference Fourier map
*R*[*F*^2^ > 2σ(*F*^2^)] = 0.066	Hydrogen site location: inferred from neighbouring sites
*wR*(*F*^2^) = 0.199	H-atom parameters not refined
*S* = 1.00	*w* = 1/[σ^2^(*F*_o_^2^) + (0.122*P*)^2^ + 2.8827*P*] where *P* = (*F*_o_^2^ + 2*F*_c_^2^)/3
2762 reflections	(Δ/σ)_max_ = 0.007
209 parameters	Δρ_max_ = 0.89 e Å^−^^3^
1 restraint	Δρ_min_ = −0.39 e Å^−^^3^

### Special details

**Table:**

Geometry. All e.s.d.'s (except the e.s.d. in the dihedral angle between two l.s. planes) are estimated using the full covariance matrix. The cell e.s.d.'s are taken into account individually in the estimation of e.s.d.'s in distances, angles and torsion angles; correlations between e.s.d.'s in cell parameters are only used when they are defined by crystal symmetry. An approximate (isotropic) treatment of cell e.s.d.'s is used for estimating e.s.d.'s involving l.s. planes.
Refinement. Refinement of *F*^2^ against ALL reflections. The weighted *R*-factor *wR* and goodness of fit *S* are based on *F*^2^, conventional *R*-factors *R* are based on *F*, with *F* set to zero for negative *F*^2^. The threshold expression of *F*^2^ > σ(*F*^2^) is used only for calculating *R*-factors(gt) *etc*. and is not relevant to the choice of reflections for refinement. *R*-factors based on *F*^2^ are statistically about twice as large as those based on *F*, and *R*- factors based on ALL data will be even larger.

### Fractional atomic coordinates and isotropic or equivalent isotropic displacement parameters (Å^2^)

**Table:**

	*x*	*y*	*z*	*U*_iso_*/*U*_eq_	
Ni1	0.5000	0.5000	0.5000	0.0219 (3)	
C1	0.7141 (7)	0.47283 (19)	0.3080 (4)	0.0293 (9)	
C2	0.5607 (7)	0.41749 (19)	0.2771 (3)	0.0310 (9)	
C3	0.3908 (6)	0.39745 (18)	0.3348 (3)	0.0250 (8)	
C4	0.2685 (7)	0.34232 (19)	0.2900 (3)	0.0287 (9)	
C5	0.0880 (8)	0.3175 (2)	0.3310 (4)	0.0380 (11)	
H5	0.0416	0.3397	0.3863	0.046*	
C6	0.0596 (7)	0.2340 (2)	0.2186 (4)	0.0310 (9)	
C7	0.3168 (7)	0.3083 (2)	0.2024 (4)	0.0333 (10)	
C8	0.5934 (9)	0.3829 (2)	0.1908 (4)	0.0448 (12)	
H8	0.7017	0.3972	0.1544	0.054*	
C9	0.5451 (11)	0.2956 (3)	0.0587 (6)	0.0655 (18)	
H9A	0.7027	0.3014	0.0599	0.079*	
H9B	0.5179	0.2510	0.0652	0.079*	
C10	0.4140 (17)	0.3190 (6)	−0.0446 (8)	0.0426 (8)	
H10A	0.2601	0.3080	−0.0501	0.168*	
H10B	0.4681	0.3005	−0.1039	0.168*	
H10C	0.4278	0.3640	−0.0473	0.168*	
C11	−0.1716 (9)	0.1431 (2)	0.2519 (4)	0.0462 (13)	
H11A	−0.2457	0.1731	0.2908	0.055*	
H11B	−0.0778	0.1164	0.3051	0.055*	
C12	−0.3438 (8)	0.1034 (2)	0.1784 (4)	0.0375 (10)	
H12A	−0.4224	0.0785	0.2233	0.045*	
H12B	−0.4517	0.1311	0.1343	0.045*	
C13	0.0646 (7)	0.1360 (2)	0.1181 (4)	0.0330 (10)	
H13A	0.1711	0.1080	0.1622	0.040*	
H13B	0.1435	0.1611	0.0735	0.040*	
C14	−0.1164 (7)	0.0976 (2)	0.0452 (4)	0.0297 (9)	
H14A	−0.2129	0.1259	−0.0039	0.036*	
H14B	−0.0469	0.0694	0.0013	0.036*	
H5N	−0.163 (7)	0.035 (2)	0.152 (3)	0.044*	
N1	0.4839 (7)	0.3299 (2)	0.1527 (3)	0.0454 (11)	
N2	0.2161 (6)	0.25401 (17)	0.1666 (3)	0.0354 (9)	
N3	−0.0183 (7)	0.26577 (19)	0.2969 (4)	0.0427 (10)	
N4	−0.0326 (6)	0.17705 (17)	0.1881 (3)	0.0327 (8)	
N5	−0.2530 (5)	0.06053 (15)	0.1053 (3)	0.0262 (7)	
O1	0.3477 (5)	0.42282 (13)	0.4188 (2)	0.0288 (7)	
O2	0.6982 (5)	0.50474 (11)	0.3906 (2)	0.0270 (7)	
O3	0.8546 (7)	0.4840 (2)	0.2525 (3)	0.0579 (11)	

### Atomic displacement parameters (Å^2^)

**Table:**

	*U*^11^	*U*^22^	*U*^33^	*U*^12^	*U*^13^	*U*^23^
Ni1	0.0256 (4)	0.0139 (4)	0.0257 (4)	−0.0008 (2)	0.0040 (3)	−0.0028 (3)
C1	0.032 (2)	0.022 (2)	0.034 (2)	−0.0029 (17)	0.0055 (18)	−0.0007 (17)
C2	0.041 (2)	0.022 (2)	0.031 (2)	−0.0069 (17)	0.0088 (18)	−0.0052 (17)
C3	0.0291 (19)	0.0178 (18)	0.026 (2)	−0.0008 (15)	0.0020 (16)	−0.0002 (16)
C4	0.034 (2)	0.023 (2)	0.030 (2)	−0.0027 (17)	0.0072 (17)	−0.0047 (17)
C5	0.040 (2)	0.032 (2)	0.046 (3)	−0.0112 (19)	0.019 (2)	−0.019 (2)
C6	0.032 (2)	0.024 (2)	0.038 (2)	−0.0061 (17)	0.0082 (18)	−0.0090 (18)
C7	0.041 (2)	0.028 (2)	0.034 (2)	−0.0039 (18)	0.0117 (19)	−0.0074 (18)
C8	0.059 (3)	0.037 (3)	0.043 (3)	−0.017 (2)	0.022 (2)	−0.012 (2)
C9	0.077 (4)	0.060 (4)	0.068 (4)	−0.025 (3)	0.034 (3)	−0.018 (3)
C10	0.0396 (18)	0.047 (2)	0.0409 (17)	0.0122 (15)	0.0083 (14)	0.0059 (15)
C11	0.052 (3)	0.041 (3)	0.053 (3)	−0.025 (2)	0.028 (2)	−0.021 (2)
C12	0.039 (2)	0.031 (2)	0.045 (3)	−0.0097 (19)	0.015 (2)	−0.011 (2)
C13	0.029 (2)	0.029 (2)	0.045 (2)	−0.0055 (17)	0.0152 (19)	−0.0153 (19)
C14	0.032 (2)	0.0219 (19)	0.037 (2)	−0.0034 (16)	0.0120 (18)	−0.0042 (17)
N1	0.061 (3)	0.040 (2)	0.042 (2)	−0.025 (2)	0.026 (2)	−0.0145 (18)
N2	0.047 (2)	0.0271 (18)	0.035 (2)	−0.0156 (16)	0.0169 (17)	−0.0112 (16)
N3	0.044 (2)	0.030 (2)	0.057 (3)	−0.0140 (17)	0.0198 (19)	−0.0192 (19)
N4	0.0348 (18)	0.0239 (18)	0.042 (2)	−0.0081 (15)	0.0143 (16)	−0.0121 (16)
N5	0.0275 (17)	0.0193 (16)	0.0303 (18)	−0.0026 (13)	0.0020 (14)	−0.0004 (14)
O1	0.0317 (15)	0.0215 (14)	0.0351 (16)	−0.0043 (11)	0.0111 (12)	−0.0069 (12)
O2	0.0329 (16)	0.0184 (14)	0.0298 (16)	0.0003 (11)	0.0066 (12)	−0.0026 (11)
O3	0.069 (3)	0.061 (2)	0.055 (2)	−0.037 (2)	0.040 (2)	−0.026 (2)

### Geometric parameters (Å, °)

**Table:**

Ni1—O2^i^	2.013 (3)	C9—C10	1.464 (12)
Ni1—O2	2.013 (3)	C9—N1	1.498 (7)
Ni1—O1^i^	2.051 (3)	C9—H9A	0.9700
Ni1—O1	2.051 (3)	C9—H9B	0.9700
Ni1—N5^ii^	2.207 (3)	C10—H10A	0.9600
Ni1—N5^iii^	2.207 (3)	C10—H10B	0.9600
C1—O3	1.236 (6)	C10—H10C	0.9600
C1—O2	1.260 (5)	C11—N4	1.472 (6)
C1—C2	1.505 (6)	C11—C12	1.509 (6)
C2—C8	1.358 (6)	C11—H11A	0.9700
C2—C3	1.448 (6)	C11—H11B	0.9700
C3—O1	1.260 (5)	C12—N5	1.480 (6)
C3—C4	1.441 (6)	C12—H12A	0.9700
C4—C7	1.398 (6)	C12—H12B	0.9700
C4—C5	1.413 (6)	C13—N4	1.450 (5)
C5—N3	1.305 (6)	C13—C14	1.522 (6)
C5—H5	0.9300	C13—H13A	0.9700
C6—N2	1.334 (6)	C13—H13B	0.9700
C6—N4	1.357 (5)	C14—N5	1.467 (5)
C6—N3	1.357 (6)	C14—H14A	0.9700
C7—N2	1.341 (6)	C14—H14B	0.9700
C7—N1	1.382 (6)	N5—Ni1^iv^	2.207 (3)
C8—N1	1.348 (6)	N5—H5N	0.90 (4)
C8—H8	0.9300		
			
O2^i^—Ni1—O2	180.0	C9—C10—H10B	109.5
O2^i^—Ni1—O1^i^	88.73 (11)	H10A—C10—H10B	109.5
O2—Ni1—O1^i^	91.27 (11)	C9—C10—H10C	109.5
O2^i^—Ni1—O1	91.27 (11)	H10A—C10—H10C	109.5
O2—Ni1—O1	88.73 (11)	H10B—C10—H10C	109.5
O1^i^—Ni1—O1	180.0	N4—C11—C12	110.6 (4)
O2^i^—Ni1—N5^ii^	90.14 (12)	N4—C11—H11A	109.5
O2—Ni1—N5^ii^	89.86 (12)	C12—C11—H11A	109.5
O1^i^—Ni1—N5^ii^	91.00 (11)	N4—C11—H11B	109.5
O1—Ni1—N5^ii^	89.00 (11)	C12—C11—H11B	109.5
O2^i^—Ni1—N5^iii^	89.86 (12)	H11A—C11—H11B	108.1
O2—Ni1—N5^iii^	90.14 (12)	N5—C12—C11	114.8 (4)
O1^i^—Ni1—N5^iii^	89.00 (11)	N5—C12—H12A	108.6
O1—Ni1—N5^iii^	91.00 (11)	C11—C12—H12A	108.6
N5^ii^—Ni1—N5^iii^	180.0	N5—C12—H12B	108.6
O3—C1—O2	122.8 (4)	C11—C12—H12B	108.6
O3—C1—C2	118.4 (4)	H12A—C12—H12B	107.6
O2—C1—C2	118.9 (4)	N4—C13—C14	110.4 (3)
C8—C2—C3	118.6 (4)	N4—C13—H13A	109.6
C8—C2—C1	116.2 (4)	C14—C13—H13A	109.6
C3—C2—C1	125.1 (4)	N4—C13—H13B	109.6
O1—C3—C4	119.5 (4)	C14—C13—H13B	109.6
O1—C3—C2	126.1 (4)	H13A—C13—H13B	108.1
C4—C3—C2	114.4 (4)	N5—C14—C13	113.6 (4)
C7—C4—C5	113.6 (4)	N5—C14—H14A	108.8
C7—C4—C3	123.4 (4)	C13—C14—H14A	108.8
C5—C4—C3	122.9 (4)	N5—C14—H14B	108.8
N3—C5—C4	124.7 (4)	C13—C14—H14B	108.8
N3—C5—H5	117.6	H14A—C14—H14B	107.7
C4—C5—H5	117.6	C8—N1—C7	118.6 (4)
N2—C6—N4	116.5 (4)	C8—N1—C9	119.9 (4)
N2—C6—N3	126.2 (4)	C7—N1—C9	121.5 (4)
N4—C6—N3	117.4 (4)	C6—N2—C7	115.9 (4)
N2—C7—N1	117.8 (4)	C5—N3—C6	115.5 (4)
N2—C7—C4	123.5 (4)	C6—N4—C13	120.5 (4)
N1—C7—C4	118.6 (4)	C6—N4—C11	122.5 (4)
N1—C8—C2	126.3 (5)	C13—N4—C11	113.0 (3)
N1—C8—H8	116.9	C14—N5—C12	108.4 (3)
C2—C8—H8	116.9	C14—N5—Ni1^iv^	113.5 (2)
C10—C9—N1	110.6 (7)	C12—N5—Ni1^iv^	115.6 (2)
C10—C9—H9A	109.5	C14—N5—H5N	109 (3)
N1—C9—H9A	109.5	C12—N5—H5N	103 (4)
C10—C9—H9B	109.5	Ni1^iv^—N5—H5N	107 (3)
N1—C9—H9B	109.5	C3—O1—Ni1	127.3 (3)
H9A—C9—H9B	108.1	C1—O2—Ni1	134.0 (3)
C9—C10—H10A	109.5		
			
O3—C1—C2—C8	1.5 (7)	N3—C6—N2—C7	5.9 (7)
O2—C1—C2—C8	−176.7 (4)	N1—C7—N2—C6	178.4 (4)
O3—C1—C2—C3	178.7 (4)	C4—C7—N2—C6	1.4 (7)
O2—C1—C2—C3	0.6 (6)	C4—C5—N3—C6	2.0 (8)
C8—C2—C3—O1	176.7 (4)	N2—C6—N3—C5	−7.5 (8)
C1—C2—C3—O1	−0.5 (7)	N4—C6—N3—C5	174.0 (4)
C8—C2—C3—C4	−1.8 (6)	N2—C6—N4—C13	11.1 (6)
C1—C2—C3—C4	−178.9 (4)	N3—C6—N4—C13	−170.2 (4)
O1—C3—C4—C7	−174.8 (4)	N2—C6—N4—C11	167.2 (4)
C2—C3—C4—C7	3.8 (6)	N3—C6—N4—C11	−14.2 (7)
O1—C3—C4—C5	5.1 (6)	C14—C13—N4—C6	−147.5 (4)
C2—C3—C4—C5	−176.4 (4)	C14—C13—N4—C11	54.4 (5)
C7—C4—C5—N3	4.1 (7)	C12—C11—N4—C6	149.6 (4)
C3—C4—C5—N3	−175.8 (5)	C12—C11—N4—C13	−52.7 (6)
C5—C4—C7—N2	−5.8 (7)	C13—C14—N5—C12	54.2 (5)
C3—C4—C7—N2	174.0 (4)	C13—C14—N5—Ni1^iv^	−176.1 (3)
C5—C4—C7—N1	177.2 (4)	C11—C12—N5—C14	−53.0 (5)
C3—C4—C7—N1	−2.9 (7)	C11—C12—N5—Ni1^iv^	178.4 (3)
C3—C2—C8—N1	−1.0 (8)	C4—C3—O1—Ni1	178.8 (3)
C1—C2—C8—N1	176.4 (5)	C2—C3—O1—Ni1	0.4 (6)
N4—C11—C12—N5	52.7 (6)	O2^i^—Ni1—O1—C3	179.7 (3)
N4—C13—C14—N5	−56.3 (5)	O2—Ni1—O1—C3	−0.3 (3)
C2—C8—N1—C7	2.0 (8)	N5^ii^—Ni1—O1—C3	89.6 (3)
C2—C8—N1—C9	−177.7 (6)	N5^iii^—Ni1—O1—C3	−90.4 (3)
N2—C7—N1—C8	−177.2 (5)	O3—C1—O2—Ni1	−178.7 (4)
C4—C7—N1—C8	0.0 (7)	C2—C1—O2—Ni1	−0.7 (6)
N2—C7—N1—C9	2.6 (8)	O1^i^—Ni1—O2—C1	−179.5 (4)
C4—C7—N1—C9	179.8 (5)	O1—Ni1—O2—C1	0.5 (4)
C10—C9—N1—C8	−89.8 (8)	N5^ii^—Ni1—O2—C1	−88.5 (4)
C10—C9—N1—C7	90.4 (7)	N5^iii^—Ni1—O2—C1	91.5 (4)
N4—C6—N2—C7	−175.6 (4)		

Symmetry codes: (i) −*x*+1, −*y*+1, −*z*+1; (ii) −*x*, *y*+1/2, −*z*+1/2; (iii) *x*+1, −*y*+1/2, *z*+1/2; (iv) −*x*, *y*−1/2, −*z*+1/2.

### Hydrogen-bond geometry (Å, °)

**Table:**

*D*—H···*A*	*D*—H	H···*A*	*D*···*A*	*D*—H···*A*
N5—H5N···O3^v^	0.90 (4)	2.29 (4)	3.161 (5)	163 (5)

Symmetry codes: (v) −*x*+1, *y*−1/2, −*z*+1/2.
